# Determinants of stunting among 0–59 months children in Lesotho: A multilevel analysis of 2023/24 Demographic and Health Survey

**DOI:** 10.1371/journal.pone.0342073

**Published:** 2026-04-06

**Authors:** Lencho Kajela Solbana, Firezer Belay Keno, Atoma Negera, Baru Legesse Kejela, Markos Desalegn, Emiru Merdassa

**Affiliations:** 1 School of Public Health, Wollega University, Nekemte, Oromia, Ethiopia; 2 Department of Nursing, Mattu University, Mattu, Oromia, Ethiopia; 3 Wollega University comprehensive specialized hospital, Wollega University, Nekemte, Oromia, Ethiopia; University of Washington, UNITED STATES OF AMERICA

## Abstract

**Background:**

Stunting is height for age below minus 2 standard deviations of the World Health Organization standard. It affected more than one-third of children in Lesotho. Few studies applied multilevel model to identify individual, household, and community-level determinants. This study aimed to assess the prevalence and multilevel determinants of stunting among children aged 0–59 months in Lesotho using data from the 2023/24 Demographic and Health Survey.

**Methods:**

We included a weighted 1,488 children aged 0–59 months across 400 enumeration areas. The weighted prevalence of stunting was determined. Individual and community level determinants of stunting were identified at a 0.05 level of significance. Models were compared using Akaike Information Criterion (AIC), Intraclass Correlation Coefficient (ICC), and Proportional Change in Variance (PCV).

**Results:**

Prevalence of stunting was 35.59% (95% CI: 33.20–38.06). Its determinants were male (AOR = 1.41; 95% CI: 1.09–1.82), age 12–23 months (AOR = 2.44; 95% CI: 1.48–4.14) and 24–59 months (AOR = 1.98; 95% CI: 1.20–3.24), 4–7 birth order (AOR = 1.43; 95% CI: 1.01–2.03), and poor (AOR = 2.58; 95% CI: 1.85–3.59) and middle wealth index (AOR = 2.03; 95% CI: 1.37–3.03). Qacha’s Nek, Thaba-Tseka, Mohale’s Hoek, and Quthing have a higher prevalence of stunting.

**Conclusion:**

35.59% of children 0–59 months in Lesotho are stunted. Its determinants were male, age 12–23 and 24–59 months, poor and middle wealth, and higher birth order. Ministry of Health, Non-governmental organizations, and community health workers should therefore strengthen age-specific, equity-driven nutrition programs.

## Introduction

Stunting, is defined as a height-for-age Z-score below minus 2 standard deviations [1,2]. It reflects the cumulative impacts of poor maternal nutrition, suboptimal infant feeding practices, recurrent illness, and inadequate environmental conditions [2,3]. In addition, it can happen due to poor environmental conditions and long-term restriction of a child's growth potential [4].

The Sustainable Development Goals (SDGs) aim to end all forms of malnutrition, including stunting, by 2030 [5]. The World Health Organization (WHO) targets a 40% reduction in stunting by 2025 [6]. Despite these international commitments, the 2023/24 Lesotho Demographic and Health Survey reveals that stunting prevalence has increased from 33% in 2014 to more than 35% in 2023/24 [7]. This places the country within the “high public health significance” range of 30–39% for stunting [4].

Stunting restricts children's adult height while increasing illness vulnerability, delaying mental growth, impairing academic performance, and even leading to early death [1,4] In 2022, 148.1 million children under five were stunted worldwide, 43% of them live in Africa [1]. Lesotho is among countries in Africa suffering from childhood stunting [7].

While previous DHS analyses have quantified stunting prevalence in Lesotho, few studies have employed multilevel models to identify both household and community-level determinants [8]. Using a recent 2023/24 Lesotho DHS data, this study examined stunting prevalence and its individual, household, and community-level determinants in children aged 0–59 months. Multilevel analysis is particularly relevant because it accounts for clustering effects and quantifies the proportion of variance attributable to community-level influences**,** which single-level models cannot capture. This approach helps disentangle whether observed disparities are due to individual circumstances or structural community differences. By identifying both levels of determinants, the analysis provides more precise and actionable evidence for policymakers, NGOs, and health professionals-allowing them to design interventions that target not only households but also community systems, thereby reducing undernutrition, morbidity, and mortality.

## Methods and materials

### Study design

A Community-based cross-sectional study design was conducted.

### Sampling design

**Fig 1:**
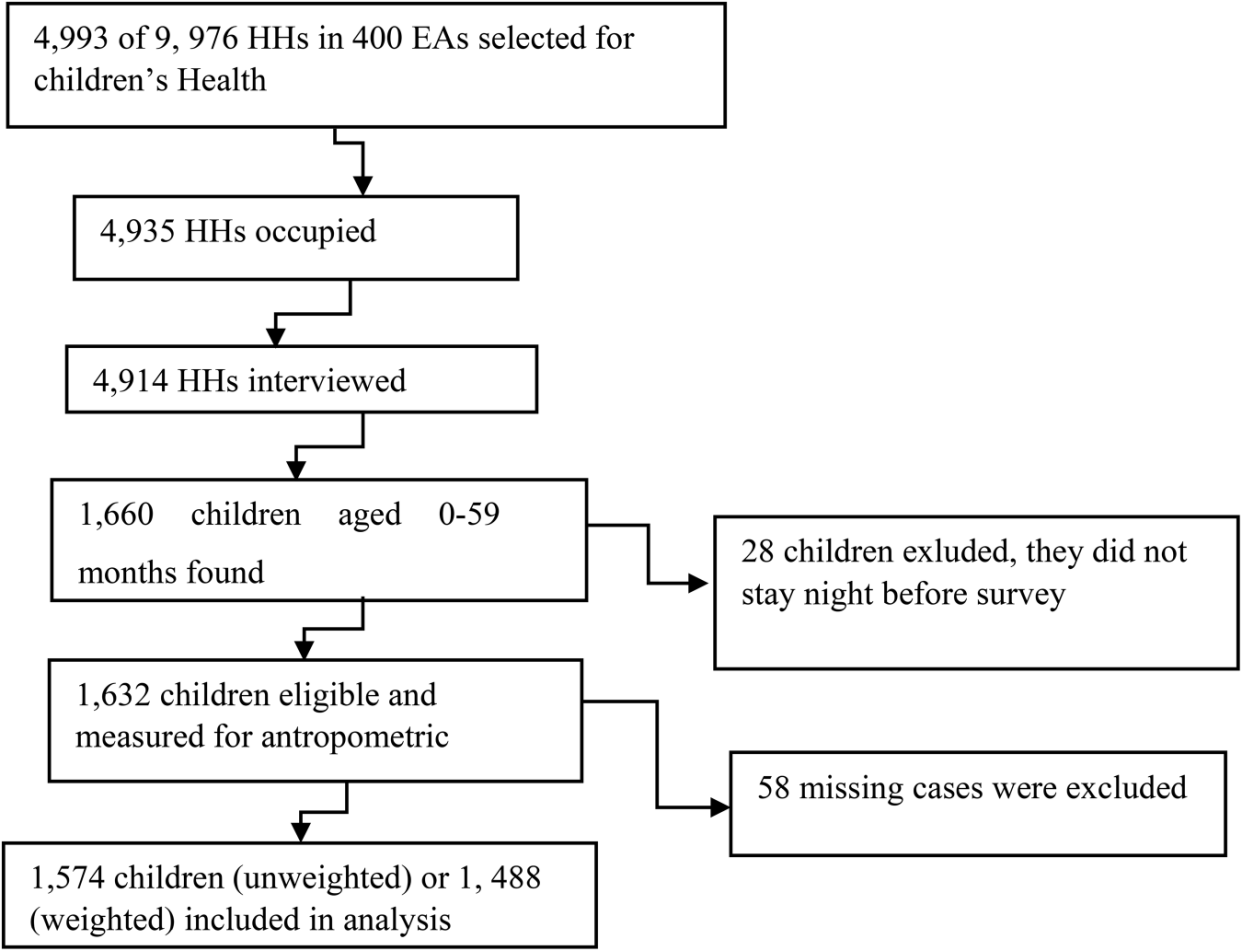
Flow diagram of sample selection.

The 2023/24 LDHS used a two-stage stratified cluster sampling design. Districts were categorized into urban, peri-urban, and rural strata. For this study, 400 enumeration areas (EAs) were selected. Next, 25 households per EA were systematically selected. Accordingly, 9,976 HHs were selected. However, 4,993 HHs were selected for children's Health Survey. After applying inclusion and exclusion criteria, 1,488 weighted participants were selected for the study Fig 1).

### Study period, area, and data source

We used data from Lesotho 2023/24 Demographic and Health Survey (LDHS). Administratively, Lesotho comprises 10 districts, each subdivided into urban, semi-urban, and rural areas.

### Population

#### Source population.

All living children aged 0–59 months in Lesotho at the time of the 2023/24 LDHS, as recorded in the survey's PR (Household Member) data file.

#### Study population.

Living children aged 0–59 months in Lesotho surveyed in the 2023/24 DHS who met the defined inclusion and exclusion criteria for numerator and denominator.

**Numerator**: Number of children aged 0–59 months whose height-for-age z-score is below minus 2 standard deviations (SD) on the WHO Child Growth Standard [4].

**Denominator**: Number of de facto living children between ages 0 and 59 months before the survey who fulfilled the following eligibility criteria.

**Inclusion criteria**

- De facto resident of Lesotho DHS 2023/24 at time of survey (hv103 = 1)
- Age between 0 and 59 months (hc1 in 0:59)
- Valid, non-flagged height-for-age Z-score (hc70 < 9990)

**Exclusion criteria**

- Missing value for height for age Z score (hc = .)
- HAZ ≤ −6.0 or ≥ + 6.0 (implausible values)

### Study variables

#### Dependent variable.

Stunting

#### Independent variables.

Both individual and community-level determinants were examined, with variable coding and descriptions presented in Table 1. Determinants were selected based on targeted literature review in Sub-Saharan Africa, ensuring inclusion of factors consistently associated with stunting [8–13]

**Table 1:** Description of independent variables.

Independent variables	Description
*Individual level factors*	
Child sex	categorized into (1) male and (2) female.
Child age	Categorized into (1) <6 months, (2) 6–11 months, (3) 12–23 months, and (4) 24–59 months.
Birth order	Categorized into (1) <4, (2) 4–7, and (3) above 7
Mother's education status	Categorized into (1) primary incomplete, (2) primary complete, and (3) secondary and above.
Household wealth index	Categorized into (1) poor, (2) middle, and (3) rich
Number of HH members aged <5 years	categorized into (1) one, (2) two, and (3) 3 and above
*Community-level factors*	
Community poverty	Categorized into (1) high poverty and (2) low poverty.
Community Education	Categorized into (1) high community education and (2) low community education.
Residence	categorized into (1) urban and (2) rural

### Operational definition

**Usual residence status** was defined by DHS variable hv103, with de jure household members (hv103 = 1) retained and all other visitors excluded.

**Child age** in completed months was defined using PR variable hc1, calculated as the difference between the interview date and the child's date of birth.

**Maternal education** was defined by PR variable hc68 and categorized as uneducated (if no education), primary (if 1–8), and secondary or higher (if ≥ 9 grade).

**Place of residence** (PR variable hv025) was defined by the household's de facto location, coded as urban (hv025 = 1) or rural (hv025 = 2).

The LDHS **wealth index** is a continuous composite score derived via principal component analysis of household asset ownership and dwelling characteristics, used to rank households from poorest to richest within the survey sample.

**Wealth index** (PR variable hv270) was categorized as poor (hv270 = poor and poorest), middle (hv270 = middle), and rich (hv270 = richer and richest).

**Community education** was defined as the cluster-level median of maternal education (PR variable hc68); clusters below the median were classified as low, and those at or above the median as high.

**Community poverty** was defined by the cluster-level proportion of households' wealth index; classified as poor, clusters with proportions below the median were categorized as low poverty, and those at or above the median as high poverty.

**De jure population:** All persons who are usual residents of the selected households, whether or not they stayed in the household the night before the interview.

**De facto population:** All persons who stayed in the selected households the night before the interview (whether usual residents or visitors).

### Measurements

Weight measurements were taken using SECA scales with a digital display (model number SECA 874U).

Height and length were measured with a ShorrBoard® measuring board. Children younger than age 24 months were measured lying down (recumbent length), while older children and adults were measured standing (height).

### Data collection procedure

Fifteen listing teams, each with three listers and a supervisor, were deployed to conduct household listing in selected enumeration areas. The DHS questionnaires were adapted from standard models to address Lesotho's specific health and population concerns. A total of 100 interviewers were recruited based on education, experience, and availability. Data collection was carried out by 15 field teams, each comprising a supervisor, three to four female interviewers, one to three male interviewers, a biomarker technician, and a driver.

### Data quality control

Household listing training took place from 28 to 30 June 2023. To verify measurement accuracy, one child per cluster was randomly selected for re-measurement. The training of trainers and pretesting were held from 28 August to 12 September, followed by fieldwork in two Berea clusters (urban and rural) from 13 to 15 September, covering 87 households. Based on the pretest, the questionnaires, translations, and CAPI system were revised. Final fieldworker training ran from 26 October to 24 November 2023.

**Data processing and analysis**

All variables were examined for outliers and missing values as part of the data cleaning process. Data analysis was conducted using Stata version 17 [14]. Categorical variables were summarized using frequencies and percentages, and results were presented in tables and charts. Sampling weights were applied to adjust for unequal selection probabilities and non-response, ensuring population-representative estimates [15]. To account for the hierarchical structure of the 2023/2024 Lesotho Demographic and Health Survey (LDHS), a two-level mixed-effects logistic regression model was employed. The model considered 1,488 (unweighted) children (level 1) nested within 400 enumeration areas (level 2)**,** allowing for adjustment of both individual- and community-level factors. Model diagnostics included the Intraclass Correlation Coefficient (ICC)**,** used to assess the proportion of total variance attributable to differences between clusters [16]. The Proportional Change in Variance (PCV) was calculated to evaluate the reduction in variance from the null model after including covariates [17]. Model selection was based on the Akaike Information Criterion (AIC)**,** with the best-fitting model identified as the one with the lowest AIC value [18]. Final results were reported as Adjusted Odds Ratios (AORs) with 95% confidence intervals (Table 2). Reporting adhered to the STROBE checklist to ensure transparent documentation of the study's methodology, findings, and implications.

**Table 2:** stunting among children characteristics and percentage distribution (2023/24 LDHS, Lesotho).

Variables	Characteristics	Stunted		COR (95% CI)
		Frequency (%)	95% CI	
Child sex	Male	300(38.28)	33.88, 42.87	1.33(1.07,1.64)
	Female	229(32.59)	28.33, 37.15	1
Child age	<6 months	36(22.45)	15.65, 31.13	1.10(0.64,1.89)
	6-11 months	25(24.29)	16.07, 36.52	1
	12-23 months	109(37.03)	30.42, 44.16	2.03(1.26,3.29)
	24-59 months	359(38.56)	34.51, 42.77	1.79(1.75,2.78)
Birth order	<4	269(31.12)	27.41, 35.08	1
	4-7	64(42.66)	32.77, 53.18	1.58(1.13,2.21)
	>7	7(53.84)	26.22, 79.28	0.99(0.36,2.70)
Wealth index	Poor	287(43.30)	39.16, 47.53	2.26(1.71, 2.98)
	Middle	105(34.23)	27.43, 41.75	1.82(1.30,2.55)
	Rich	137(26.52)	21.06, 32.81	1
Maternal education	Primary incomplete	46(35.86)	26.83, 46.02	1
	Primary complete	87(46.77)	38.82, 54.87	1.35(0.90,2.02)
	Secondary and above	254(30.26)	26.30, 34.54	0.76(0.54,1.07)
Number of <5 children	1	290(32.59)	28.72, 36.72	1
	2	158 (37.44)	31.54, 43.75	1.22(0.96,1.55)
	3 and above	82(46.32)	37.00, 55.90	1.72(1.24,2.39)
Residence	Urban	143(29.09)	23.87, 34.94	
	Rural	387(38.79)	35.04, 42.69	1.46(1.12,1.88)
Community poverty	High	222(40.86)	36.73, 45.13	1.46(1.16,1.82)
	Low	308(32.56)	28.35, 37.08	1
Community education	High	245(32.59)	28.10, 37.42	1
	Low	272(39.02)	34.74, 43.48	1.40(1.12,1.176)

### Handling missing values

Children who were not weighed or measured, or whose height values were not recorded, were excluded from both the numerator and denominator. In addition, any child records with out-of-range or invalid z-scores were identified and removed from both the numerator and denominator to maintain data integrity and ensure valid prevalence estimates [15,19].

### Ethical consideration

We obtained an ethical approval letter for this secondary analysis from the DHS program IRB, and all authors had full access to the data. This study analyzed publicly available Demographic and Health Survey (DHS) data on child stunting in accordance with the Declaration of Helsinki. Original data collection under the DHS protocol was reviewed and approved by the DHS S&T IRB. Field teams secured oral informed consent from every woman respondent and, when applicable, from parents or legal guardians before interviews and anthropometric measurements, with each consent documented by the interviewer's signature on a dated form; no written consent was sought. Because the DHS data are fully de-identified and publicly accessible, our secondary analysis was deemed exempt from further ethical review, and the requirement for new consent was waived (S1 File)

## Results

### Prevalence of stunting

Prevalence of stunting was 35.59% (95% CI 33.20–38.06). It was 38.28% in males and 32.59% in females, peaking at 38.56% among those aged 24–59 months. In addition, it was was 43.30% in the poor and 26.53% in the rich Households (Table 2).

### Prevalence of stunting across districts

**Fig 2:**
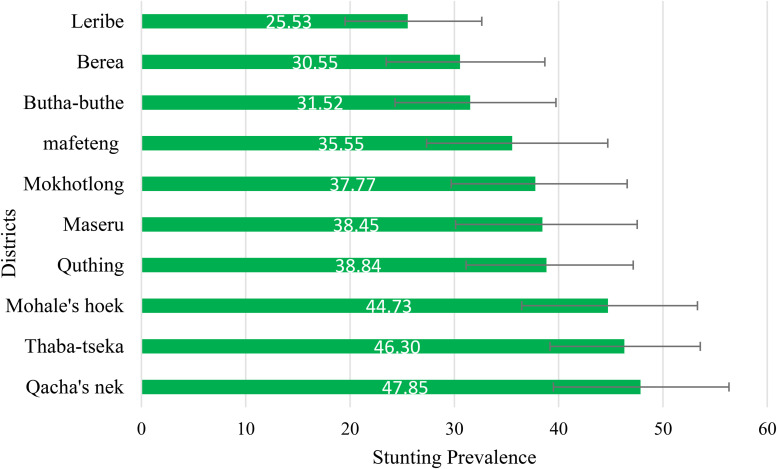
District‐level prevalence of stunting among children aged 0–59 months in Lesotho 2023/24 DHS.

In this study, the district-level prevalence of stunting varied substantially. It ranges from 25.53% in Leribe to 47.85% in Qacha’s Nek, with a range of 22.32% (Fig 2).

### Multilevel logistic regression analysis

Males had 1.41 times higher odds of stunting compared to females. Children aged 12–23 months and 24–59 months had 2.44 times and 1.98 times higher odds of stunting, respectively, compared to those aged 6–11 months. Compared with birth order < 4, birth order 4–7 was associated with 1.43 times higher odds of stunting. Children from poor and middle households had 2.58 times and 2.03 times higher odds of stunting, respectively, than those from rich households (Table 3).

**Table 3:** Multilevel analysis of determinants of stunting among 0–59 months children in Lesotho.

Variable	Category	Null model	Individual-level determinants	Community-level determinants	Individual+community determinants
		AOR (95% CI)	AOR (95% CI), p-value	AOR (95% CI), p-value	AOR (95% CI), p-value
Child sex	Male		1.42(1.09,1.83),.008		**1.41(1.09,1.82),.**009*
	Female		1		1
Child age	<6 months		1.21(0.68,2.17),.518		1.22(0.68,2.19),.500
	6-11 months		1		1
	12-23 months		2.39(1.41,4.05),.001		**2.44(1.48,4.14),.**001*
	24-59 months		1.94(1.18, 3.19),.009		**1.98(1.20,3.24),.**007*
Birth order	<4		1		1
	4-7		1.47(1.03,2.12),.036		**1.43(1.01,2.03),.**043*
	>7		0.85(0.30,2.42),.756		0.80(0.28,2.24),.666
Wealth index	Poor		2.54(1.79,3.59),.000		**2.58(1.85,3.59),.**000*
	Middle		2.02(1.36,3.01).001		**2.03(1.37,3.03),.**000*
	Rich		1		1
Maternal education	Primary incomplete		1		
	Primary complete		1.42(0.92, 2.21),.117		
	Secondary and above		1.16(0.77,1.74),.473		
Number of <5 children	1		1		
	2		1.32(0.99,1.76),.062		
	3 and above		1.16(0.76,1.76),.486		
Residence	Urban				
	Rural			1.24(0.92,1.67),0.166	
Community poverty	High			1.20(0.91,1.58),.192	
	Low			1	
Community education	High			1	
	Low			1.23(0.96,1.58),.099	
Variance		.1422665	2.42e-15	.1046861	2.85e-11
ICC%		4.15	<0.01	1.2	<0.01
PCV %			~100%	26.4%	~100%
**Model fit statistics**		**Model I**	**Model II**	**Model III**	**Model IV**
AIC		2086.01	1389.5	2037.196	1387.94

*Significant at 0.05, AIC: Akaike Information Criterion AOR: Adjusted odds ratio:Bold cell: significant, ICC: Intraclass Correlation Coefficient

Statistically significant between-community variation in the null model justified using a multilevel mixed-effects approach, with clusters accounting for about 4% of the total stunting variance (ICC). After adding individual and community predictors, the final model fully explained this variance (PCV = 100%). Model comparison by AIC identified Model V as the best fit (Table 3).

## Discussion

This study found that 35.59% of children aged 0–59 months in Lesotho are stunted. It is higher than WHO’s Global Nutrition Target of a 40% reduction in under-five stunting by 2025 [20]. Despite Lesotho’s initial progress of reducing stunting from 39% in 2004 to 33% in 2014 [7], it rose back to 35.59% in 2023/24. This finding may suggest a reversal of earlier nutritional interventions. From 2004 to 2014, Lesotho’s government and partners implemented a National Multisectoral Nutrition Strategy integrating district services, community malnutrition management, universal supplementation, breastfeeding support, and school feeding. These interventions reduced stunting by 70% [21]. Further research is needed to pinpoint the demographic, socioeconomic, environmental, and health-system factors driving this reversal.

In Lesotho, Qacha’s Nek (47.9%), Thaba-Tseka (46.3%), and Mohale’s Hoek (44.7%) exhibit the highest prevalence of childhood stunting. These disparities may reflect localized challenges in achieving food security and accessing healthcare and nutrition services, as highlighted by recent national surveys and food insecurity analyses. Therefore, further efforts should be strengthened to improve food security and address other underlying causes of stunting in Lesotho, in order to reduce the burden of childhood stunting in these areas [22,23]. In addition, further district-level research is required to assess food security, health-service access, socio-economic factors and environmental stressors driving these disparities.

Males had 1.41 times higher odds of stunting compared to females. This is supported by studies done in Indonesia [24], Nigeria [25], Afghanistan [11], and DHS based meta-analysis in SSA [13]. Males have a higher probability of diarrhea and respiratory infection [12,26,27]. There is also evidence that males are less likely to initiate exclusive breastfeeding [28] and are more likely to initiate complementary feeding before 6 months than females [12]. The other evidence indicates that males have higher energy requirement than females [10]. These could increase the odds of stunting among males. Strengthening community education on equitable feeding practices could help close it.

Children aged 12–23 months and 24–59 months had 2.44 times and 1.98 times higher odds of stunting than those aged 6–11 months, respectively. This is in line with studies in Ethiopia [29], Afghanistan [11], and systematic reviews [10,30]. There is evidence that this age group is susceptible to diarrheal disease, intestinal parasite and other acute infections [9], which might hinder appetite and food absorption. The other possible justification can be related to the discontinuation of breastfeeding and introduction of other feeding after 1 year. This may compromise child growth, lower immunity and contributes infection [10]. Furthermore, on some occasions, women may return to work during this time, where child is left with older children or another person.

This study found that children with a birth order of 4–7 had 1.43 times higher odds of stunting compared to <4. Studies from Afghanistan [11] and SSA [31,32] support this finding. In Lesotho, the average fertility rate is 2.5 children per woman, with 17% of teenage girls experiencing pregnancy. Higher birth order may relate to larger family size and shorter interpregnancy intervals. Contraceptive use among currently married women stands at 67% [7]. This might increase the risk of stunting due to lack of food for entire family, especially among poor families [10]. Targeted reproductive Health and nutrition program that promotes adequate birth spacing and strengthens caregiving support for later-born children [33].

Furthermore, children from poor and middle wealth households had 2.58 times and 2.03 times higher odds of stunting compared to rich, respectively. Previous studies support this finding [9–11]. Having higher wealth improves access to healthcare for mother and child [10]. In addition, poorer households lack access to diverse and nutritious foods, clean water, sanitation, and quality healthcare, all of which are essential for healthy child growth [34].

The finding that more than one-third of children under five in Lesotho are stunted highlights a critical gap in achieving both SDG 2: Zero Hunger by 2030 and the WHO Global Nutrition Targets for 2025. Urgent, coordinated action to strengthen food security, improve maternal and child nutrition, and scale up community-based interventions is essential if Lesotho is to accelerate progress and safeguard the health and development of its youngest generation [5,6].

### Strength and limitations

This study used data from the recent nationally representative 2023/24 Lesotho Demographic and Health Survey, enhancing the generalizability of its findings. Nonetheless, it has several limitations:

- The cross-sectional design precludes causal inference.
- Potential recall bias may affect self-reported variables.
- Key behavioural and cultural determinants were not included.
- District-level drivers of stunting disparities were not explored.

## Conclusion

Stunting remains high at 35.59%, and its determinants were age 12–23 and 24–59 months, male, 4–7 birth order, and poor and middle wealth index.

### Recommendations

**Ministry of Health should**:

- Strengthen early childhood nutrition by expanding community-based complementary feeding support (6–24 months) and scaling up growth monitoring with age-appropriate feeding for children 12–59 months.
- Integrate family-planning and nutrition services by offering birth-spacing counseling alongside complementary-feeding support for mothers of four or more children.

**Non-governmental organizations (NGOs) should:**

- Deliver targeted nutrition support to poor and middle income households.
- Offer microloans and vocational training to boost household income, and organize mother-support groups on affordable, locally sourced meal planning.

**Community health workers should:**

- Strengthen regular home visits to monitor growth among children 0–59 months and flag stunting risk, prioritizing boys, high birth‐order siblings, those aged 12–59 months, and children from poor and middle‐wealth households.
- Deliver tailored nutrition counselling and demonstrating complementary feeding with affordable, locally available foods.

**Future researchers should:**

- Conduct district-level studies in areas like Qacha’s Nek and Thaba-Tseka to identify local barriers and design context-specific interventions.
- Undertake longitudinal investigations to address this study's limitations and clarify causal pathways in child stunting.

## Supporting information

S1 File PLOS One Human Subjects Research Checklist. (PDF)

## Abbreviations

DHS: Demographic and Health Survey
WHO: World Health Organization
IRB: Institutional review board
AIC: Akaike Information Criterion
ICC: Intraclass Correlation Coefficient
